# *N*-(4-Methyl­phen­yl)-6-(pyrazol-1-yl)pyridazin-3-amine

**DOI:** 10.1107/S1600536810024700

**Published:** 2010-07-03

**Authors:** Abdul Qayyum Ather, M. Nawaz Tahir, Misbahul Ain Khan, Muhammad Makshoof Athar, Eliana Aparecida Silicz Bueno

**Affiliations:** a Department of Chemistry, Islamia University, Bahawalpur, Pakistan, Applied Chemistry Research Center, PCSIR Laboratories Complex, Lahore 54600, Pakistan; b Department of Physics, University of Sargodha, Sargodha, Pakistan; c Department of Chemistry, Islamia University, Bahawalpur, Pakistan; d Institute of Chemistry, University of the Punjab, Lahore, Pakistan; e Instituto de Quimica, Universidade Estadual de Londrina, Londrina, Pr., Brazil

## Abstract

In the title compound, C_14_H_13_N_5_, the pyrazole ring is disordered over two orientations in a 0.571 (10):0.429 (10) ratio and the dihedral angle between the pyridazine ring and the benzene ring is 28.07 (10)°. In the crystal, pairs of N—H⋯N and C—H⋯N hydrogen bonds link the mol­ecules into dimers, with the aid of a crystallographic twofold axis. The packing is consolidated by further C—H⋯N bonds and weak C—H⋯π inter­actions.

## Related literature

For related structures, see: Ather *et al.* (2009 ▶, 2010*a* ▶,*b* ▶,*c* ▶). For graph-set notation, see: Bernstein *et al.* (1995 ▶).
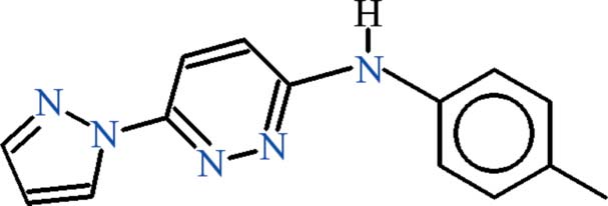


## Experimental

### 

#### Crystal data

C_14_H_13_N_5_
*M* *_r_* = 251.29
Monoclinic, 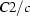

*a* = 31.8677 (17) Å
*b* = 7.9408 (5) Å
*c* = 10.8446 (7) Å
β = 109.715 (3)°
*V* = 2583.4 (3) Å^3^
*Z* = 8
Mo *K*α radiation
μ = 0.08 mm^−1^
*T* = 296 K
0.32 × 0.18 × 0.16 mm

#### Data collection

Bruker Kappa APEXII CCD diffractometer
Absorption correction: multi-scan (*SADABS*; Bruker, 2005 ▶) *T*_min_ = 0.982, *T*_max_ = 0.988
9293 measured reflections
2336 independent reflections
1384 reflections with *I* > 2σ(*I*)
*R*_int_ = 0.059

#### Refinement

*R*[*F* ^2^ > 2σ(*F* ^2^)] = 0.060
*wR*(*F* ^2^) = 0.176
*S* = 1.03
2336 reflections
156 parameters
11 restraints
H-atom parameters constrained
Δρ_max_ = 0.40 e Å^−3^
Δρ_min_ = −0.33 e Å^−3^

### 

Data collection: *APEX2* (Bruker, 2007 ▶); cell refinement: *SAINT* (Bruker, 2007 ▶); data reduction: *SAINT*; program(s) used to solve structure: *SHELXS97* (Sheldrick, 2008 ▶); program(s) used to refine structure: *SHELXL97* (Sheldrick, 2008 ▶); molecular graphics: *ORTEP-3 for Windows* (Farrugia, 1997 ▶) and *PLATON* (Spek, 2009 ▶); software used to prepare material for publication: *WinGX* (Farrugia, 1999 ▶) and *PLATON*.

## Supplementary Material

Crystal structure: contains datablocks global, I. DOI: 10.1107/S1600536810024700/hb5514sup1.cif

Structure factors: contains datablocks I. DOI: 10.1107/S1600536810024700/hb5514Isup2.hkl

Additional supplementary materials:  crystallographic information; 3D view; checkCIF report

## Figures and Tables

**Table 1:** Hydrogen-bond geometry (Å, °) *Cg*1 is the centroid of the N4/N5*B*/C12*B*–C14*B* ring.

*D*—H⋯*A*	*D*—H	H⋯*A*	*D*⋯*A*	*D*—H⋯*A*
N1—H1⋯N2^i^	0.86	2.12	2.982 (3)	178
C6—H6⋯N3^i^	0.93	2.62	3.498 (4)	157
C14*B*—H14*B*⋯N5*B*^ii^	0.93	2.47	3.357 (11)	160
C5—H5⋯*Cg*1^iii^	0.93	2.99	3.527 (5)	118

Symmetry codes: (i) 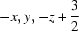; (ii) 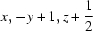; (iii) 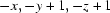.

**Fig. 1.**
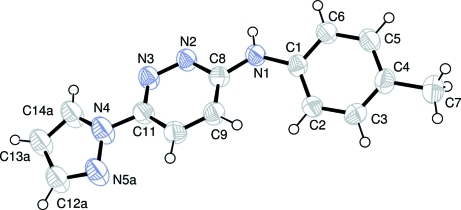
View of (I) showing the major orientation of the pyrazole ring with displacement ellipsoids drawn at the 50% probability level. H-atoms are shown as small spheres of arbitrary radii.

**Fig. 2.**
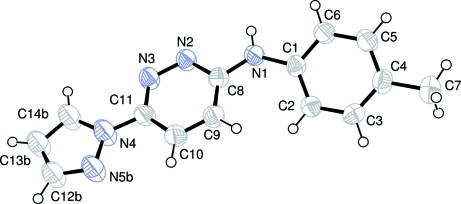
View of (I) showing the minor orientation of the pyrazole ring with displacement ellipsoids drawn at the 50% probability level. H-atoms are shown as small spheres of arbitrary radii.

**Fig. 3.**
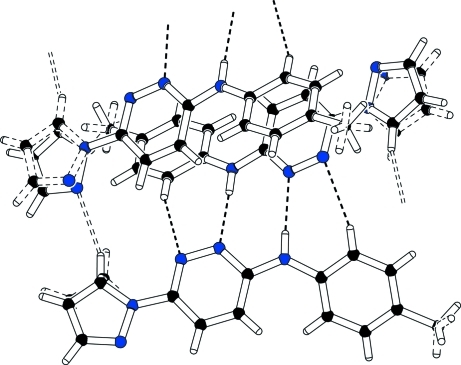
Packing diagram of (I), showing that the dimers are formed with ring motifs.

## supplementary crystallographic information

### Comment

In continuation to pyrazolylpyridazine derivatives (Ather *et al.*, 2009, 2010*a*, 2010*b*, 2010*c*), the title compound (I, Fig. 1) is being reported here.

The title compound is reaction product of 3-chloro-6-(1*H*-pyrazol-1-yl)pyridazine and 4-toluidine. There are three cyclic rings in the final product. In (I), the pyrazole ring except adjoining N-atom and H-atoms of the only methyl group are disordered over two set of sites with occupancy ratio of 0.571 (10):0.429 (10). The majority group A (N4/N5B/C12B/C13B/C14B), the pyridazine ring B (C8—C11/N3/N2) and the 4-toludine group C (N1/C1—C7) are planar with r. m. s. deviations of 0.0431, 0.0175 and 0.0153 Å respectively. The miniority disordered group D (N4/N5A/C12A/C13A/C14A) is also planar with r. m. s. deviation of 0.0555 Å. The dihedral angle between A/B, A/C and B/C is 6.46 (24)°, 32.15 (28)° and 28.07 (10)° respectively. The dihedral angle between the disordered groups A/D is 17.72 (34)°. There exist intermolecular H-bondings of N—H···N and C—H···N types (Table 1). The molecules are stabilized in the form of dimers. In dimers, one ring motif of *R*_2_^2^(8) and two ring motifs of *R*_2_^2^(7) types (Bernstein *et al.*, 1995) are present (Fig. 2). C—H···π interaction (Table 1) also play role in stabilizing the molecules.

### Experimental

3-Chloro-6-(1*H*-pyrazol-1-yl)pyridazine (1.68 g, 9.33 mmol) and 4-toluidine (1 g, 9.34 mmol) were refluxed in dimethylformamide (DMF) for 2 h. The reaction mixture was concentrated under vacuum and poured in cold water. The precipitates obtained were filtered, washed with distilled water and dried to give 74.0% yield. The product obtained was purified by column chromatography and recrystallized in ethanol to afford light brown needles of title compound (I).

### Refinement

The bond distances, bond angles and thermal elipsoids present in the pyrazol ring showed that there is disorder. Similarly difference Fourier map showed that H-atoms of methyl are also disordered. For the disordered heavy atoms the bond distances and bond angles are best fitted according to the known structures (Ather *et al.*, 2009, 2010a,b,c). The disordered atoms were refined using equal anisotropic thermal parameters.

The H-atoms were positioned geometrically (N–H = 0.86, C–H = 0.93–0.96 Å) and refined as riding with *U*_iso_(H) = x*U*_eq_(C, N), where x = 1.5 for methyl and x = 1.2 for all other H-atoms.

### Crystal data

**Table:**

C_14_H_13_N_5_	*F*(000) = 1056
*M**_r_* = 251.29	*D*_x_ = 1.292 Mg m^−^^3^
Monoclinic, *C*2/*c*	Mo *K*α radiation, λ = 0.71073 Å
Hall symbol: -C 2yc	Cell parameters from 1384 reflections
*a* = 31.8677 (17) Å	θ = 2.7–25.2°
*b* = 7.9408 (5) Å	µ = 0.08 mm^−^^1^
*c* = 10.8446 (7) Å	*T* = 296 K
β = 109.715 (3)°	Needle, light brown
*V* = 2583.4 (3) Å^3^	0.32 × 0.18 × 0.16 mm
*Z* = 8	

### Data collection

**Table:**

Bruker Kappa APEXII CCD diffractometer	2336 independent reflections
Radiation source: fine-focus sealed tube	1384 reflections with *I* > 2σ(*I*)
graphite	*R*_int_ = 0.059
Detector resolution: 7.5 pixels mm^-1^	θ_max_ = 25.2°, θ_min_ = 2.7°
ω scans	*h* = −38→38
Absorption correction: multi-scan (*SADABS*; Bruker, 2005)	*k* = −9→9
*T*_min_ = 0.982, *T*_max_ = 0.988	*l* = −11→13
9293 measured reflections	

### Refinement

**Table:**

Refinement on *F*^2^	Primary atom site location: structure-invariant direct methods
Least-squares matrix: full	Secondary atom site location: difference Fourier map
*R*[*F*^2^ > 2σ(*F*^2^)] = 0.060	Hydrogen site location: inferred from neighbouring sites
*wR*(*F*^2^) = 0.176	H-atom parameters constrained
*S* = 1.03	*w* = 1/[σ^2^(*F*_o_^2^) + (0.0773*P*)^2^ + 1.8092*P*] where *P* = (*F*_o_^2^ + 2*F*_c_^2^)/3
2336 reflections	(Δ/σ)_max_ < 0.001
156 parameters	Δρ_max_ = 0.40 e Å^−^^3^
11 restraints	Δρ_min_ = −0.32 e Å^−^^3^

### Special details

**Table:**

Geometry. Bond distances, angles *etc*. have been calculated using the rounded fractional coordinates. All su's are estimated from the variances of the (full) variance-covariance matrix. The cell e.s.d.'s are taken into account in the estimation of distances, angles and torsion angles
Refinement. Refinement of *F*^2^ against ALL reflections. The weighted *R*-factor *wR* and goodness of fit *S* are based on *F*^2^, conventional *R*-factors *R* are based on *F*, with *F* set to zero for negative *F*^2^. The threshold expression of *F*^2^ > σ(*F*^2^) is used only for calculating *R*-factors(gt) *etc*. and is not relevant to the choice of reflections for refinement. *R*-factors based on *F*^2^ are statistically about twice as large as those based on *F*, and *R*- factors based on ALL data will be even larger.

### Fractional atomic coordinates and isotropic or equivalent isotropic displacement parameters (Å^2^)

**Table:**

	*x*	*y*	*z*	*U*_iso_*/*U*_eq_	Occ. (<1)
N1	−0.01706 (7)	0.2747 (3)	0.5655 (2)	0.0510 (9)	
N2	0.04995 (8)	0.3448 (3)	0.7127 (2)	0.0510 (10)	
N3	0.09382 (8)	0.3783 (4)	0.7500 (2)	0.0524 (10)	
N4	0.15970 (9)	0.4068 (4)	0.7102 (3)	0.0707 (8)	
N5B	0.18195 (19)	0.4362 (12)	0.6217 (6)	0.0707 (8)	0.571 (10)
C1	−0.05015 (9)	0.2122 (4)	0.4533 (3)	0.0442 (10)	
C2	−0.04181 (10)	0.1187 (4)	0.3561 (3)	0.0505 (11)	
C3	−0.07680 (10)	0.0626 (4)	0.2491 (3)	0.0530 (11)	
C4	−0.12061 (10)	0.0918 (4)	0.2371 (3)	0.0549 (11)	
C5	−0.12852 (10)	0.1783 (5)	0.3377 (3)	0.0559 (13)	
C6	−0.09415 (9)	0.2382 (4)	0.4439 (3)	0.0509 (11)	
C7	−0.15858 (7)	0.0306 (5)	0.1201 (2)	0.0777 (14)	
C8	0.02676 (6)	0.3091 (3)	0.58716 (18)	0.0442 (10)	
C9	0.04727 (6)	0.3151 (3)	0.49129 (18)	0.0533 (11)	
C10	0.09141 (10)	0.3458 (4)	0.5291 (3)	0.0572 (13)	
C11	0.11362 (9)	0.3742 (4)	0.6618 (3)	0.0502 (11)	
C12B	0.2222 (2)	0.4805 (14)	0.6947 (7)	0.0707 (8)	0.571 (10)
C13B	0.2269 (2)	0.4969 (12)	0.8208 (8)	0.0707 (8)	0.571 (10)
C14B	0.1867 (3)	0.4636 (14)	0.8260 (9)	0.0707 (8)	0.571 (10)
C13A	0.2274 (3)	0.4340 (16)	0.8474 (10)	0.0707 (8)	0.429 (10)
C14A	0.1844 (4)	0.4271 (19)	0.8420 (11)	0.0707 (8)	0.429 (10)
N5A	0.1847 (3)	0.3606 (15)	0.6367 (9)	0.0707 (8)	0.429 (10)
C12A	0.2262 (3)	0.3897 (18)	0.7145 (9)	0.0707 (8)	0.429 (10)
H3	−0.07056	0.00336	0.18333	0.0638*	
H7D	−0.16231	0.10527	0.04753	0.1168*	0.571 (10)
H5	−0.15778	0.19657	0.33365	0.0670*	
H6	−0.10053	0.29660	0.50978	0.0610*	
H9	0.03074	0.29820	0.40341	0.0639*	
H10	0.10641	0.34781	0.46897	0.0688*	
H12B	0.24530	0.49867	0.66181	0.0847*	0.571 (10)
H13B	0.25249	0.52537	0.88974	0.0847*	0.571 (10)
H14B	0.17843	0.47764	0.89986	0.0847*	0.571 (10)
H7E	−0.18551	0.02840	0.14120	0.1168*	0.571 (10)
H7F	−0.15208	−0.08070	0.09709	0.1168*	0.571 (10)
H1	−0.02583	0.29429	0.63089	0.0611*	
H2	−0.01262	0.09367	0.36274	0.0605*	
H7A	−0.17227	−0.06543	0.14465	0.1168*	0.429 (10)
H7B	−0.14740	−0.00041	0.05142	0.1168*	0.429 (10)
H7C	−0.18023	0.11882	0.08976	0.1168*	0.429 (10)
H12A	0.25110	0.38307	0.68855	0.0847*	0.429 (10)
H13A	0.25231	0.46082	0.91929	0.0847*	0.429 (10)
H14A	0.17366	0.43441	0.91163	0.0847*	0.429 (10)

### Atomic displacement parameters (Å^2^)

**Table:**

	*U*^11^	*U*^22^	*U*^33^	*U*^12^	*U*^13^	*U*^23^
N1	0.0412 (13)	0.073 (2)	0.0373 (15)	−0.0006 (13)	0.0111 (11)	−0.0051 (13)
N2	0.0433 (14)	0.068 (2)	0.0398 (15)	−0.0029 (13)	0.0117 (11)	0.0001 (12)
N3	0.0446 (14)	0.070 (2)	0.0410 (15)	−0.0049 (13)	0.0122 (12)	0.0027 (13)
N4	0.0481 (9)	0.106 (2)	0.0581 (12)	−0.0103 (12)	0.0181 (8)	0.0075 (13)
N5B	0.0481 (9)	0.106 (2)	0.0581 (12)	−0.0103 (12)	0.0181 (8)	0.0075 (13)
C1	0.0422 (16)	0.052 (2)	0.0352 (16)	−0.0011 (14)	0.0090 (13)	0.0037 (14)
C2	0.0428 (16)	0.060 (2)	0.0476 (19)	0.0018 (15)	0.0137 (14)	−0.0025 (16)
C3	0.057 (2)	0.060 (2)	0.0432 (18)	−0.0024 (16)	0.0186 (15)	−0.0079 (16)
C4	0.0505 (18)	0.066 (2)	0.0430 (19)	−0.0077 (16)	0.0088 (15)	−0.0011 (16)
C5	0.0421 (17)	0.071 (3)	0.054 (2)	−0.0001 (16)	0.0155 (15)	−0.0004 (17)
C6	0.0459 (17)	0.066 (2)	0.0407 (18)	0.0012 (16)	0.0145 (14)	0.0004 (15)
C7	0.057 (2)	0.101 (3)	0.066 (2)	−0.013 (2)	0.0088 (18)	−0.019 (2)
C8	0.0447 (16)	0.047 (2)	0.0388 (17)	0.0043 (14)	0.0112 (13)	0.0033 (14)
C9	0.0527 (18)	0.071 (2)	0.0351 (17)	−0.0060 (16)	0.0133 (14)	−0.0021 (15)
C10	0.0534 (19)	0.076 (3)	0.0466 (19)	−0.0070 (17)	0.0226 (15)	−0.0046 (17)
C11	0.0452 (17)	0.064 (2)	0.0416 (18)	−0.0003 (15)	0.0148 (14)	0.0035 (16)
C12B	0.0481 (9)	0.106 (2)	0.0581 (12)	−0.0103 (12)	0.0181 (8)	0.0075 (13)
C13B	0.0481 (9)	0.106 (2)	0.0581 (12)	−0.0103 (12)	0.0181 (8)	0.0075 (13)
C14B	0.0481 (9)	0.106 (2)	0.0581 (12)	−0.0103 (12)	0.0181 (8)	0.0075 (13)
C13A	0.0481 (9)	0.106 (2)	0.0581 (12)	−0.0103 (12)	0.0181 (8)	0.0075 (13)
C14A	0.0481 (9)	0.106 (2)	0.0581 (12)	−0.0103 (12)	0.0181 (8)	0.0075 (13)
N5A	0.0481 (9)	0.106 (2)	0.0581 (12)	−0.0103 (12)	0.0181 (8)	0.0075 (13)
C12A	0.0481 (9)	0.106 (2)	0.0581 (12)	−0.0103 (12)	0.0181 (8)	0.0075 (13)

### Geometric parameters (Å, °)

**Table:**

N1—C1	1.405 (4)	C12A—C13A	1.472 (14)
N1—C8	1.363 (3)	C12B—C13B	1.331 (11)
N2—N3	1.344 (4)	C13A—C14A	1.353 (17)
N2—C8	1.342 (3)	C13B—C14B	1.328 (12)
N3—C11	1.312 (4)	C2—H2	0.9300
N4—N5B	1.392 (7)	C3—H3	0.9300
N4—C11	1.407 (4)	C5—H5	0.9300
N4—C14B	1.339 (10)	C6—H6	0.9300
N4—N5A	1.354 (10)	C7—H7D	0.9600
N4—C14A	1.389 (12)	C7—H7E	0.9600
N5A—C12A	1.327 (14)	C7—H7F	0.9600
N5B—C12B	1.308 (10)	C7—H7A	0.9600
N1—H1	0.8600	C7—H7B	0.9600
C1—C6	1.387 (4)	C7—H7C	0.9600
C1—C2	1.386 (4)	C9—H9	0.9300
C2—C3	1.384 (4)	C10—H10	0.9300
C3—C4	1.377 (5)	C12A—H12A	0.9300
C4—C5	1.382 (5)	C12B—H12B	0.9300
C4—C7	1.508 (4)	C13A—H13A	0.9300
C5—C6	1.379 (4)	C13B—H13B	0.9300
C8—C9	1.403 (3)	C14A—H14A	0.9300
C9—C10	1.348 (4)	C14B—H14B	0.9300
C10—C11	1.391 (4)		
			
C1—N1—C8	130.3 (2)	C1—C2—H2	120.00
N3—N2—C8	120.5 (2)	C3—C2—H2	120.00
N2—N3—C11	118.9 (2)	C2—C3—H3	119.00
N5B—N4—C11	119.0 (4)	C4—C3—H3	119.00
N5B—N4—C14B	105.9 (6)	C4—C5—H5	119.00
C11—N4—C14B	132.2 (5)	C6—C5—H5	119.00
N5A—N4—C11	118.6 (5)	C1—C6—H6	120.00
C11—N4—C14A	124.3 (6)	C5—C6—H6	120.00
N5A—N4—C14A	113.3 (7)	C4—C7—H7D	109.00
N4—N5A—C12A	103.7 (8)	C4—C7—H7E	109.00
N4—N5B—C12B	104.6 (5)	C4—C7—H7F	109.00
C8—N1—H1	115.00	C4—C7—H7A	109.00
C1—N1—H1	115.00	C4—C7—H7B	109.00
C2—C1—C6	118.2 (3)	C4—C7—H7C	109.00
N1—C1—C6	117.1 (3)	H7D—C7—H7E	109.00
N1—C1—C2	124.6 (3)	H7D—C7—H7F	109.00
C1—C2—C3	120.2 (3)	H7E—C7—H7F	110.00
C2—C3—C4	122.0 (3)	H7A—C7—H7B	110.00
C3—C4—C5	117.3 (3)	H7A—C7—H7C	109.00
C5—C4—C7	121.1 (3)	H7B—C7—H7C	109.00
C3—C4—C7	121.7 (3)	C8—C9—H9	121.00
C4—C5—C6	121.7 (3)	C10—C9—H9	121.00
C1—C6—C5	120.6 (3)	C9—C10—H10	121.00
N2—C8—C9	120.7 (2)	C11—C10—H10	121.00
N1—C8—C9	125.82 (18)	C13A—C12A—H12A	125.00
N1—C8—N2	113.5 (2)	N5A—C12A—H12A	125.00
C8—C9—C10	118.7 (2)	C13B—C12B—H12B	123.00
C9—C10—C11	117.3 (3)	N5B—C12B—H12B	123.00
N4—C11—C10	121.2 (3)	C14A—C13A—H13A	128.00
N3—C11—C10	123.8 (3)	C12A—C13A—H13A	127.00
N3—C11—N4	114.9 (3)	C14B—C13B—H13B	128.00
N5A—C12A—C13A	110.8 (9)	C12B—C13B—H13B	128.00
N5B—C12B—C13B	113.3 (7)	C13A—C14A—H14A	127.00
C12A—C13A—C14A	104.9 (9)	N4—C14A—H14A	127.00
C12B—C13B—C14B	104.4 (7)	C13B—C14B—H14B	125.00
N4—C14A—C13A	105.3 (9)	N4—C14B—H14B	125.00
N4—C14B—C13B	110.6 (8)		
			
C8—N1—C1—C2	23.6 (5)	N1—C1—C2—C3	180.0 (3)
C8—N1—C1—C6	−160.4 (3)	C6—C1—C2—C3	3.9 (5)
C1—N1—C8—N2	−171.0 (3)	N1—C1—C6—C5	−179.0 (3)
C1—N1—C8—C9	11.4 (4)	C2—C1—C6—C5	−2.7 (5)
C8—N2—N3—C11	−0.5 (4)	C1—C2—C3—C4	−2.2 (5)
N3—N2—C8—N1	178.7 (2)	C2—C3—C4—C5	−0.8 (5)
N3—N2—C8—C9	−3.5 (4)	C2—C3—C4—C7	179.9 (3)
N2—N3—C11—N4	−178.5 (3)	C3—C4—C5—C6	2.1 (5)
N2—N3—C11—C10	3.6 (5)	C7—C4—C5—C6	−178.6 (3)
C11—N4—N5B—C12B	173.1 (6)	C4—C5—C6—C1	−0.4 (5)
C14B—N4—N5B—C12B	10.1 (10)	N1—C8—C9—C10	−178.0 (3)
N5B—N4—C11—N3	−168.2 (5)	N2—C8—C9—C10	4.6 (4)
N5B—N4—C11—C10	9.8 (6)	C8—C9—C10—C11	−1.7 (4)
C14B—N4—C11—N3	−10.4 (8)	C9—C10—C11—N3	−2.4 (5)
C14B—N4—C11—C10	167.5 (7)	C9—C10—C11—N4	179.8 (3)
N5B—N4—C14B—C13B	−11.5 (10)	N5B—C12B—C13B—C14B	−1.2 (13)
C11—N4—C14B—C13B	−171.3 (6)	C12B—C13B—C14B—N4	8.0 (12)
N4—N5B—C12B—C13B	−5.6 (12)		

### Hydrogen-bond geometry (Å, °)

**Table:**

Cg1 is the centroid of the N4/N5B/C12B–C14B ring.

**Table:**

*D*—H···*A*	*D*—H	H···*A*	*D*···*A*	*D*—H···*A*
N1—H1···N2^i^	0.86	2.12	2.982 (3)	178
C6—H6···N3^i^	0.93	2.62	3.498 (4)	157
C14B—H14B···N5B^ii^	0.93	2.47	3.357 (11)	160
C5—H5···Cg1^iii^	0.93	2.99	3.527 (5)	118

Symmetry codes: (i) −*x*, *y*, −*z*+3/2; (ii) *x*, −*y*+1, *z*+1/2; (iii) −*x*, −*y*+1, −*z*+1.
